# Effect of interaction of external surfaces on the symmetry and lattice distortion of CdSe nanocrystals by molecular dynamics simulations

**DOI:** 10.1007/s11051-017-4090-5

**Published:** 2017-12-03

**Authors:** S. Stelmakh, K. Skrobas, S. Gierlotka, B. Palosz

**Affiliations:** Institute of High Pressure Physics PAS, ul. Sokolowska 29/37, 01-142 Warsaw, Poland

**Keywords:** CdSe, Nanocrystals, Molecular dynamics, Surface structure, Bond length gradient, Modeling and simulation

## Abstract

The effect of interaction of low-index atomic planes, (100), (110), and (111) terminating CdSe platelet nanocrystals is examined using molecular dynamics (MD) simulations. Asymmetry of the environment of atoms at the end surface layers leads to anisotropic deformation of the cubic lattice and to a relative shift of Cd and Se sub-lattices. Interference of distortions of the crystal lattice originating at the terminal surfaces leads to changes of symmetry of the CdSe lattice in the whole sample volume. In the models, 2–3 nm thick, for all types of surfaces under examination, the initial cubic lattice symmetry gets lost in the whole sample volume.

**Graphical abstract:**
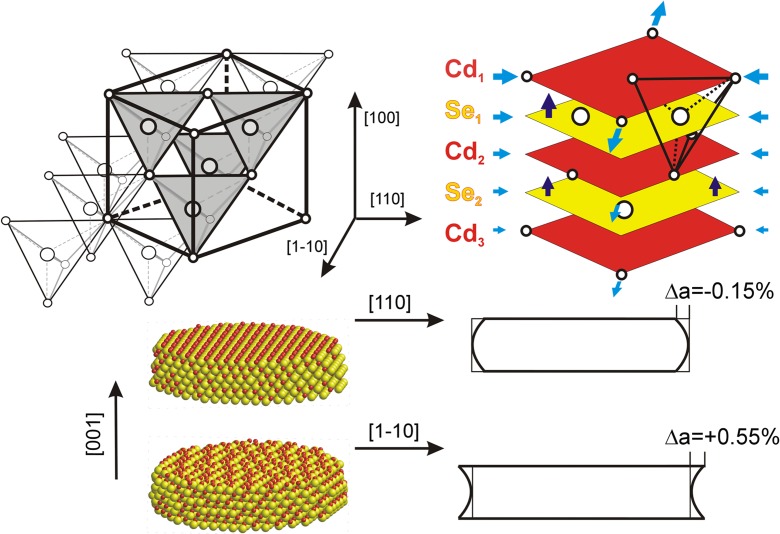
ᅟ

ᅟ

## Introduction

In studies on unique properties of nanocrystals, the real crystal structure of an individual nano-grain is very important since a complete picture of the physical properties of nanocrystals requires information about their atomic structure. Such information was not really available until recently due to a lack of reliable information about the actual atomic arrangements at the surface and inside the nanocrystal grain. Our ultimate goal is to learn about the “whole” atomic structure of nanocrystals, i.e., about the atomic architecture of the surfaces terminating a single nanocrystalline grain and on the internal lattice underneath the surface, using CdSe as the model material.

Since the best available experimental techniques either lack the required resolution (HRTEM), or can only visualize atoms at the very surface (STM, AFM), modeling and computer simulation is practically the only way to obtain information on the atomic structure of nanocrystals with resolution required to examine the symmetry and short- and long-range atomic arrangements in the crystals.

The rearrangement of atoms at the surface occurs due to the environment of the atoms located at the crystal terminating planes, which is always different than that in the crystal volume. Recently, we presented the results of molecular dynamics (MD) simulations of models of CdSe crystal platelets about 6 nm thick with basal planes being the atomic planes with low Miller indices of (100), (110), or (111). We showed that the changes in the symmetry and the lengths of interatomic bonds at the surface layer have a pronounced effect on the positions of atoms underneath the surface. The corresponding changes in interatomic lattice distances are observed up to 3 nm below the surface, beyond which the parent (bulk) crystal lattice remains unaffected. This borderline depth can be called the relaxation length of the surface strain (RL). In crystals with sizes comparable to surface relaxation length the strains appearing at the end surfaces (their magnitude depending on the atomic planes terminating the crystal) will obviously interact with each other, i.e., the changes of positions of atoms underneath the terminating surfaces will overlap (Stelmakh et al. 2017). This effect can appear in nanocrystals with only a few nanometers in size. In such crystals, the atomic lattice throughout the entire grain volume is determined by the type of surfaces terminating the crystal volume and their spacing. There are multiple experimental reports on the size dependence of lattice parameters of semiconductor nanocrystals, including CdSe (Tolbert and Alivisatos 1995; Zhang et al. 2002; Masadeh et al. 2007). The proposed explanations usually recall surface tension (Zhang et al. 2002) and the resulting internal pressure as being responsible for the effect. In the present work, we show that the actual situation is by far more complex. The internal structure of the nanocrystals is neither uniform nor isotropic and the observed changes of the apparent average lattice parameters are caused by the crystal lattice rearrangement that originates at the surface and extends into the bulk of nanocrystals.

## Computations

### MD simulations

The molecular dynamics (MD) technique is a widely used methodology of computer simulation of the structure and properties of materials on the atomic scale (Rapaport 2004; Griebel et al. 2007). The method requires far less computer power than the ab initio quantum mechanical methods and therefore allows for modeling the actual bulk structures (Car and Parrinello 1985). While ab initio approach is used for calculations of electronic band structure, vibrational properties of nanocrystals are routinely being modeled with molecular dynamics methods using the classical Newtonian equations of motion.

Several potentials that reproduce well bulk lattice parameter, bulk modulus, specific heat, and phonon spectra of CdSe have been proposed (Benkabou et al. 2000; Rabani 2001; Han and Bester 2011). Their applicability to nanoparticles has recently been analyzed (Kelley 2016) and the long-known Tersoff formula (Tersoff 1988) parametrized by Benkabou et al.(Benkabou et al. 2000) was found to reproduce the experimental data on Raman scattering, infrared spectra, and phonon dispersion for both small (approximately 400 atoms) and large (approximately 2600 atoms) nanocrystals in the best way. The Tersoff potential (TP) is designated for short ranged, covalent bonds. It is a function of 11 empirical parameters. It takes into account the local environment of atoms, i.e., the angle between bonds and the distances between atoms, and their population. The TP decreases with an increase in the number of interacting neighbors, which allows to distinguish between bulk and surface atoms if their interatomic distances are similar.

In the present study, the simulations were run with the POLY-classic software using high-end PC-class computer (Todorov et al. 2006). Parameters of the Tersoff potential were taken from Benkabou where it is shown that the bulk lattice parameter is reproduced to within 0.08% (Benkabou et al. 2000). Although TP works very well in the crystal’s bulk, its capability of accurate reproduction of the surface structure is limited. It is short-range and accounts only for the interaction of the nearest-neighbor atoms. It can reproduce 2 × 1 surface reconstruction but no reconstruction with longer periodicity can be obtained. For the same reason, surface diffusion cannot be reliably modeled. Yet, it produces reasonable figures for the surface energy. Our simulations of small CdSe nanoparticles (not the subject of the present report) yielded the following values of surface energy for low-index atomic surfaces: 2.18 eV/atom for (100), 2.11 eV/atom for (110), 1.78 eV/atom for (111)A, and 2.40ev/atom for (111)B surfaces. This is in general agreement with the experimental values reported in the literature (Xu et al. 2011). The differences between various surfaces naturally reflect the density of the broken atomic bonds at the surface.

The input models were generated by NanoPDF64 software code (Skrobas 2017). The same simulation protocol was applied to all models; during the first 10,000 steps (10 ps), the system was warmed up to relax the initial configuration. Then the system was simulated in the Hoover-Nose thermostat/barostat for 20,000 steps (20 ps). During simulation, the temperature was kept at *T* = 300 K and the pressure at *P* = 1 atm. We examined models build with up to 20,000 Cd and Se atoms arranged in the zinc-blende crystal lattice.

Being aware of the limitations of TP in the present work, we do not discuss the surface features but analyze the crystal structure deeper in the bulk.

From the simulation data, we calculated the vibrational density of states function VDOS as a Fourier transform of velocity autocorrelation function VAF (Goncalves and Bonadeo 1992). In our models, the highest frequency vibrational modes appear at about 6–7 THz; therefore, the characteristic period of thermal vibrations is about 0.15 ps. To evaluate the averaged (equilibrium) results of the simulations, the atomic coordinates of atoms in the examined models were taken as averages in the final 10 ps of each simulation run.

### MD-simulated models

MD simulations were performed for cylindrical models of 12 nm in diameter. The cylinders were approximately 2.5 nm thick so that the distance between the terminating planes is comparable to the relaxation length of the surface strains previously determined for thick CdSe cylinders (Stelmakh et al. 2017). In this work, models terminated by the same low-index atomic terminal planes, (100), (110), (111)A, and (111)B, are examined. Note that we discern between two types of (111) surfaces; in the case of the (111)A terminal plane, the surface layer is spaced from the underlying layer of the other sub-lattice by ¼ *c*_0_, while at the surface of type (111)B, the top atomic layer is three times further away, i.e., it is distant by ¾ *c*_0_, c.f. section “Symmetry and disordering of the surface layers”.

The effect of interaction between terminating planes is examined through analysis of changes of a few shortest interatomic distances characterizing the near-neighbor coordination: *r*_2_ which is the shortest distance within sub-lattices (Cd-Cd, Se-Se) and *r*_1_ and *r*_3_ which describe relative positions of Cd and Se sub-lattices (Cd-Se). The lattice distortion was measured both in the planes and between the neighboring atomic planes parallel to the surface. Since the relaxation at the edges and the lateral surfaces of the simulated cylindrical particle may interfere with the examined changes occurring at and beneath the basal planes, only the atoms located in the central, 6 nm in diameter, section of the model were taken into account.

The average lengths of *r*_1_, *r*_2_, and *r*_3_ were determined from the reduced pair distribution function *G*(*r*) calculated for a fraction of atoms selected from the MD models (Egami and Billinge 2003). Those atoms were either from a given atomic plane, from a string of atoms, or from pairs or triples of subsequent atomic planes.

The effect of interaction of two opposite parallel surfaces was evaluated by comparison to the effects determined previously for 6-nm thick models (Stelmakh et al. 2017).

## Results and discussion

### Symmetry and disordering of the surface layers

In our recent study performed for CdSe cylinders built from 20 to 26 CdSe layers, the average values of interatomic distances were measured inside individual atomic planes parallel to the surface and also between adjacent atomic planes (Stelmakh et al. 2017). Possible anisotropy of the lattice deformation was not accounted for and therefore changes of the symmetry were not analyzed. We found that, among four different surface types under examination, the end layers of (100) and (111)B surfaces lose the long range ordering and the surface adopts a disordered structure. At the (110) and (111)A surfaces, long-range order and the symmetry are preserved (Stelmakh et al. 2017). In the present work, we examine anisotropy of changes of the interatomic distances which occurs at the surface and which may affect the symmetry of the internal crystal lattice.

#### Symmetry of (100) surface

**Fig. 1:**
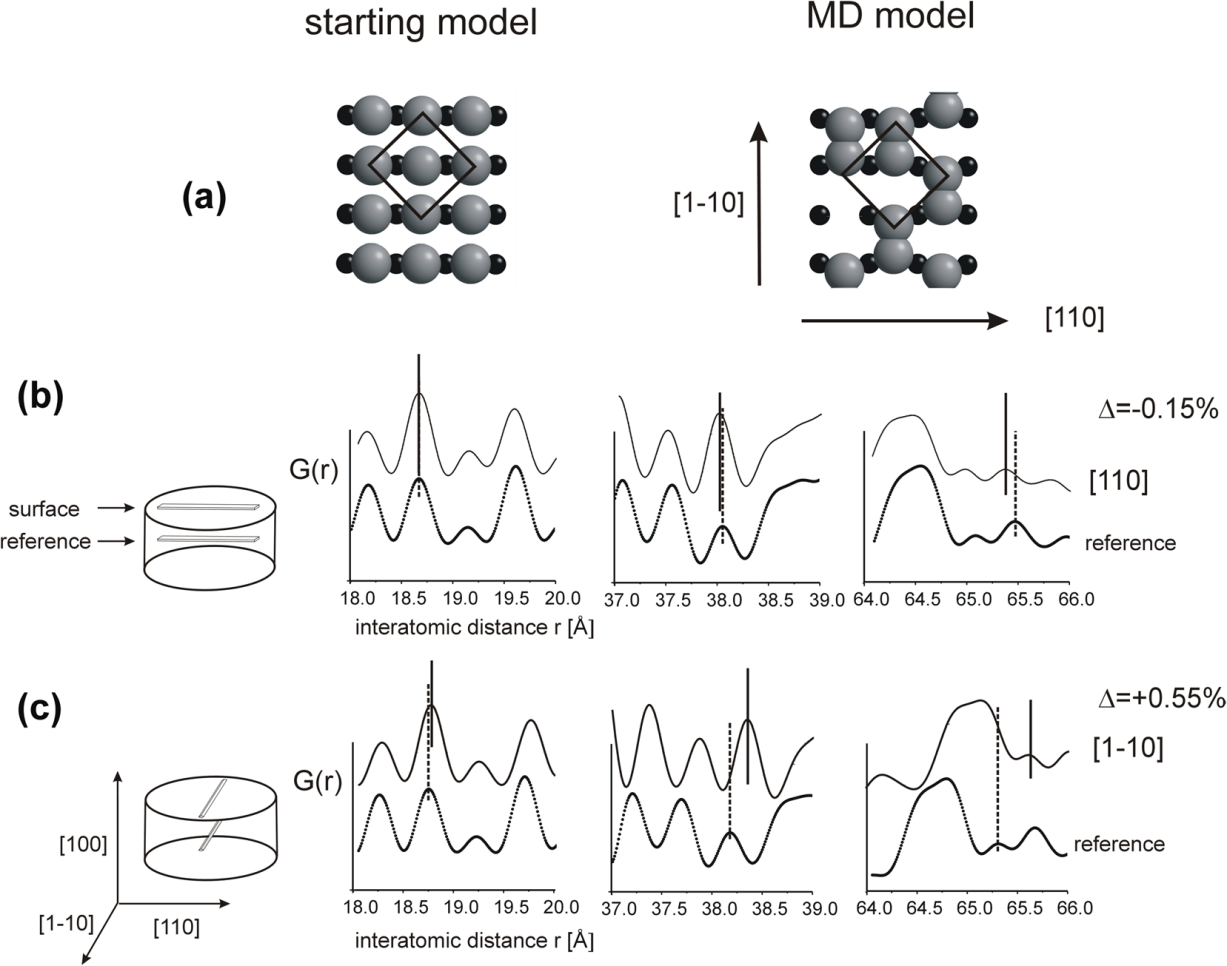
**a** The first two atomic layers at the (100) CdSe surface before and after MD simulation, **b** interatomic distance function *G*(*r*) calculated in the [110] and, **c** in the [1–10] directions

A model of CdSe with (100) surface is built from monoatomic layers, alternately Cd and Se; the end layer is either Cd or Se, the layer underneath Se or Cd, respectively. Cd and Se lattices are displaced relative to each other by ¼ of the diagonal of the unit square, in the [110] or [1–10] direction (Fig. 1a). Since the atoms of the sub-lattice underneath the end layer are aligned in the [110] direction, the end surface atoms have a possibility to move in the [1–10] direction with a choice of two equivalent alternate positions. They have no such choice in the [110] direction since each end atom is strongly bonded to two atoms of the next layer. As a result the end atoms form randomly distributed pairs and the original fourfold symmetry at the terminating (100) atomic planes is lost.

Calculations of *G*(*r*) functions were made for sets of atoms “cut out” from three atomic surface layers (excluding the end layer due to disordering). The sets were 1 nm wide and 10 nm long in two equivalent perpendicular directions, [110] and [1–10]. A comparison to the perfect lattice shows that in-plane distances are shortened by 0.15% in the [110] direction (Fig. 1b) and are elongated by 0.55% in the [1–10] direction (Fig. 1c); the square arrangement of atoms in the layers underneath the disordered surface is transformed into orthorhombic one.

**Fig. 2:**
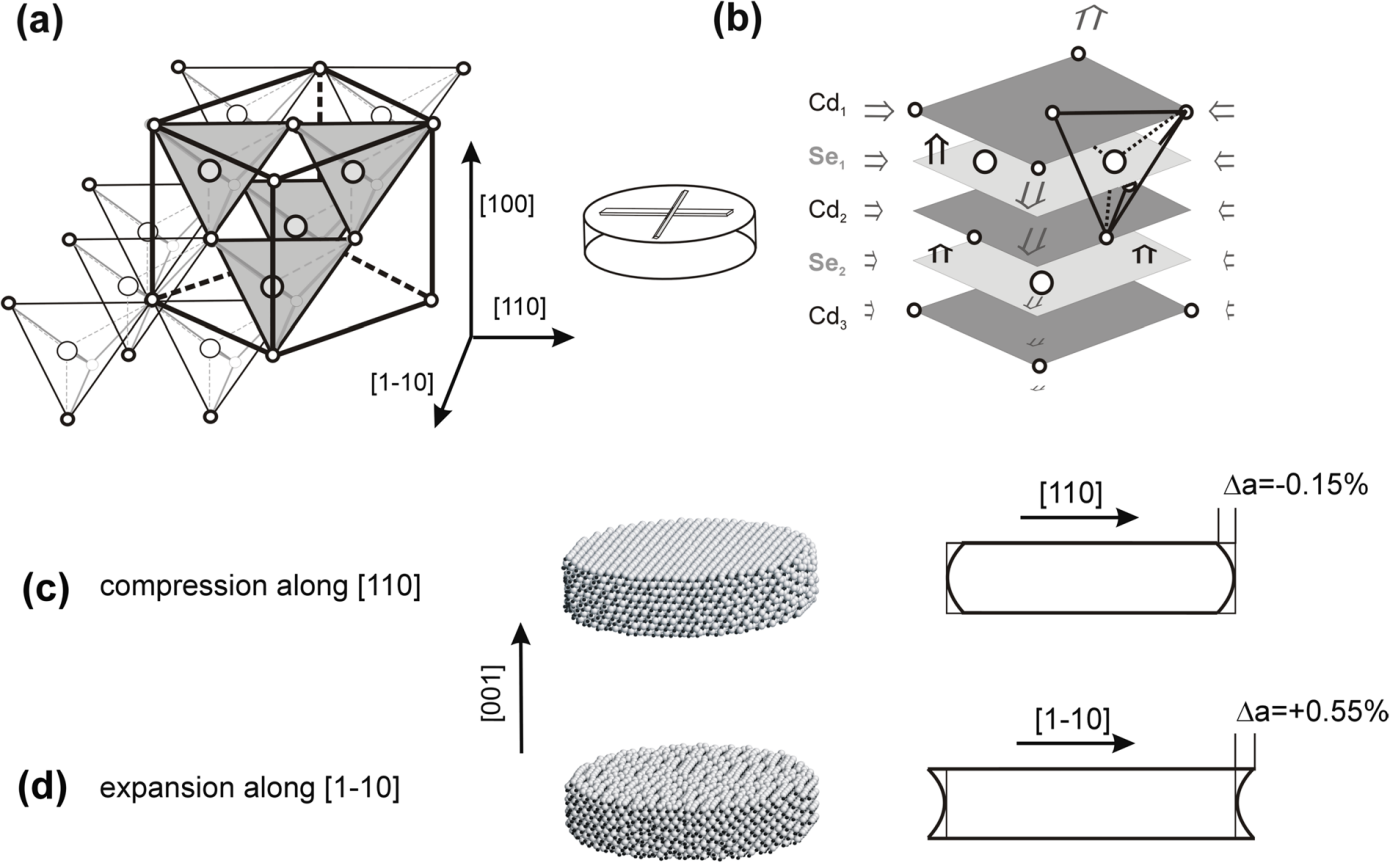
Deformation of CdSe cubic lattice at the (100) surface

In-plane surface deformation is accompanied by a relative shift of Cd and Se sub-lattices by about 1% in the [001] direction (Fig. 2b). This shift decreases with an increase in the distance from the surface, c.f. section “Interaction of (100) surfaces”. In previously examined 6-nm thick models, the cubic lattice is fully recovered at the depth of about 2 nm, but in the models built of only nine layers, the cubic symmetry is lost in the whole volume (Stelmakh et al. 2017). The effect of the surface deformation is demonstrated in Fig. 2(c, d) which show that, as a result of the surface deformation, the lines of atoms perpendicular to the surface (in the [001] direction) bend, and they bend differently in the (110) than in the (1-10) atomic planes.

#### Symmetry of (110) surface

**Fig. 3:**
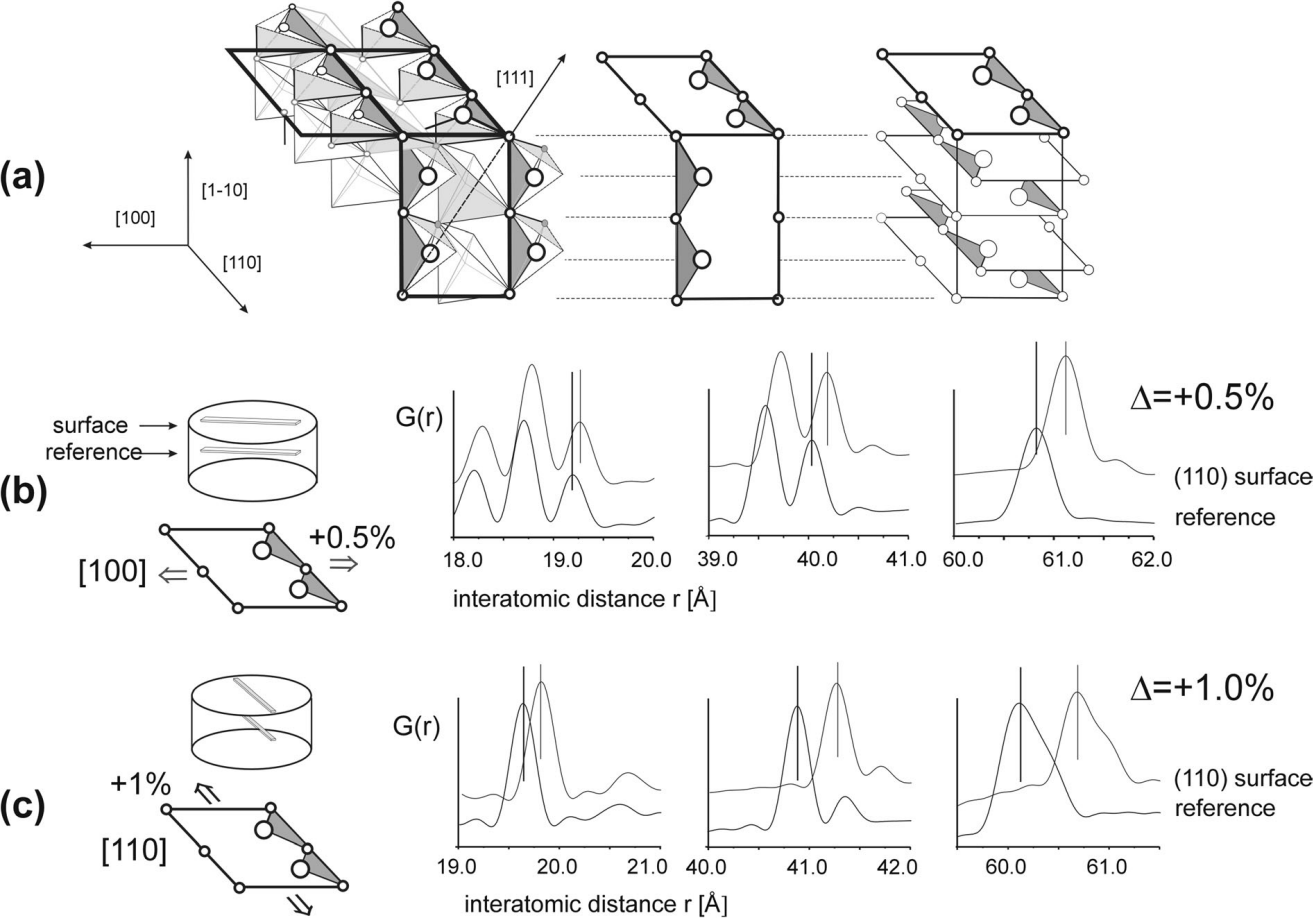
**a** A model of the atomic structure of CdSe terminated by the (110) surface. b and **c** The lattice deformation in MD simulated models and interatomic distance functions *G*(*r*) calculated for the [100] and [110] directions, respectively

The surface (110) is bi-atomic Cd and Se sub-lattices being shifted in the plane relative to each other by ¼ of the diagonal of the cubic unit cell ([111] direction) (Fig. 3):

The calculations of the *G*(*r*) functions showed that, at the surface layer, the interatomic distances are elongated by 0.5% in the [100] and by 1% in the [110] direction (Fig. 3) and the fourfold symmetry of the surface is lost. Lattice deformation at the very surface is the same in thick and thin models. In the thick model, the deformation disappears at the depth of about 2 nm, while in the thin model extends thru the entire volume.

#### Symmetry of (111)A surface

**Fig. 4:**
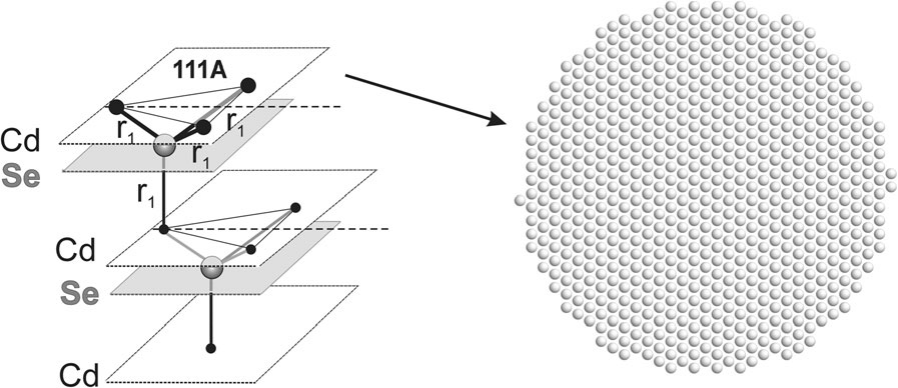
A sequence of hexagonal Cd and Se layers underneath the (111)A surface and a projection of the end Cd layer after MD simulation

For (111) planes, the environment of each individual atom at the end monoatomic layers for both (111)A and (111)B surfaces is identical; therefore, no deformation of individual hexagonal layers is expected. At the (111)A plane, the long range order is preserved; each individual atom at this surface (Cd or Se) is strongly bonded to three atoms (Se or Cd) of the next hexagonal layer (being at the distance *r*_1_), which stabilizes the hexagonal surface layer (Fig. 4). The surface strain is demonstrated only by a changed length of interatomic bonds, both in the plane and between individual planes, c.f. section “Interaction of (111)A surfaces”.

#### Symmetry of (111)B surface

**Fig. 5:**
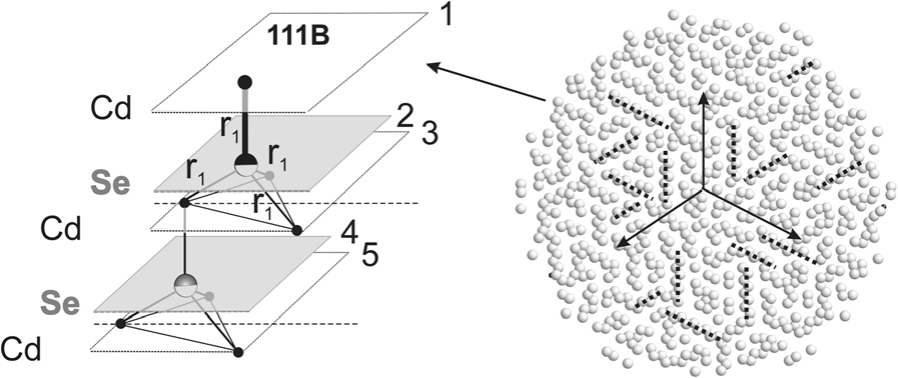
The sequence of hexagonal Cd and Se layers underneath the (111)A surface and a projection of the end Cd layer after MD simulation

At the (111)B surface, the threefold symmetry is lost and replaced by a strong disorder, resembling an amorphous-like pattern with only short-range order being well-defined (Stelmakh et al. 2017). At that, surface each atom of the end layer (Cd or Se) is bonded strongly to only one atom of the layer underneath it (Se or Cd) by the Cd-Se bond of length *r*_1_ (Fig. 5). The next nearest neighbor bonds of the end surface atoms are much weaker and have the length of *r*_3_ in the equivalent directions corresponding to the original threefold symmetry of the initial model, c.f. section “Interaction of (111)B surfaces”.

During relaxation of the end surface layer, the individual atoms rearrange and form chains of the same atoms, where each pair of the atoms, Cd-Cd or Se-Se, is equally distant. The long axis of each chain is oriented in one of three possible directions which follow the original threefold lattice symmetry, but their lengths and relative orientation are not correlated (Fig. 5).

**Fig. 6:**
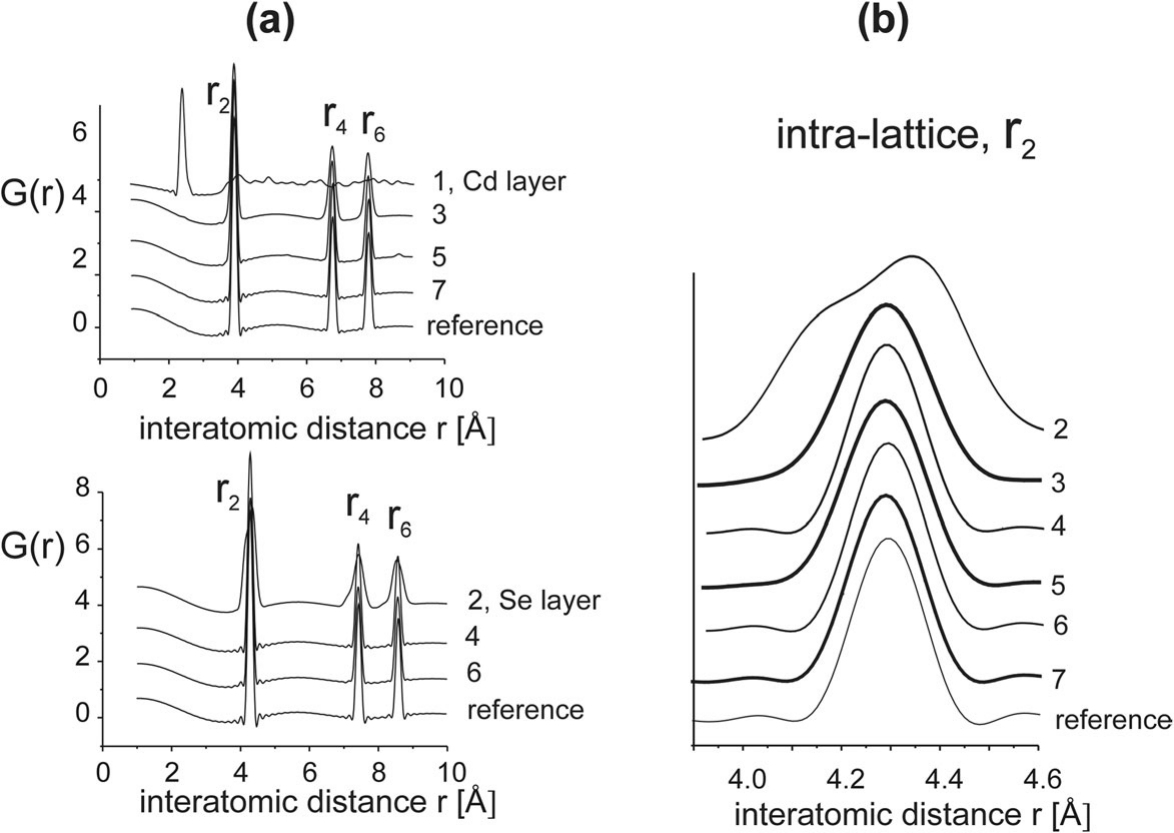
**a** Interatomic distance functions *G*(*r*) of individual Cd and Se layers underneath the (111)B end surface. **b** *G*(*r*) lines of Cd-Cd and Se-Se *r*_2_ distances calculated for subsequent layers increasingly distant from the surface

Due to a lack of long-range ordering at the end atomic layer, a disorder appears also in the crystal lattice underneath. Figure 6a presents partial *G*(*r*) functions calculated for subsequent Cd and Se layers at and under the surface. *G*(*r*) of the Cd end surface (layer 1) has one strong peak at about 2.5 Å, which corresponds to the bonds between Cd-Cd atoms arranged in the chains; the length of the bonds is about the same as *r*_1_ of the Cd-Se bonds in the CdSe crystal lattice. In the Se layer underneath it (layer 2), a split of *r*_2_ distances is observed, and in the subsequent Cd and Se layers, the width of *r*_2_ peaks systematically decreases with an increase in the distance from the surface. However, even the width of the *r*_2_, peak of the seventh layer is larger than that in the reference CdSe lattice. That means that positional disordering extends to the depth larger than 2 nm.

Detailed comparison of *r*_2_ peaks (Fig. 6b) shows that the width of the peak calculated for a Cd layer is always larger than that for the subsequent Se layer. Since broadening of peaks in *G*(*r*) is a measure of a dispersion of positions of atoms about their average values, the present result indicates that the disordering that appears at the Cd end layer extends to the other layers unevenly: Cd layers inside the grain volume show stronger positional disorder than the Se layers.

**Fig. 7:**
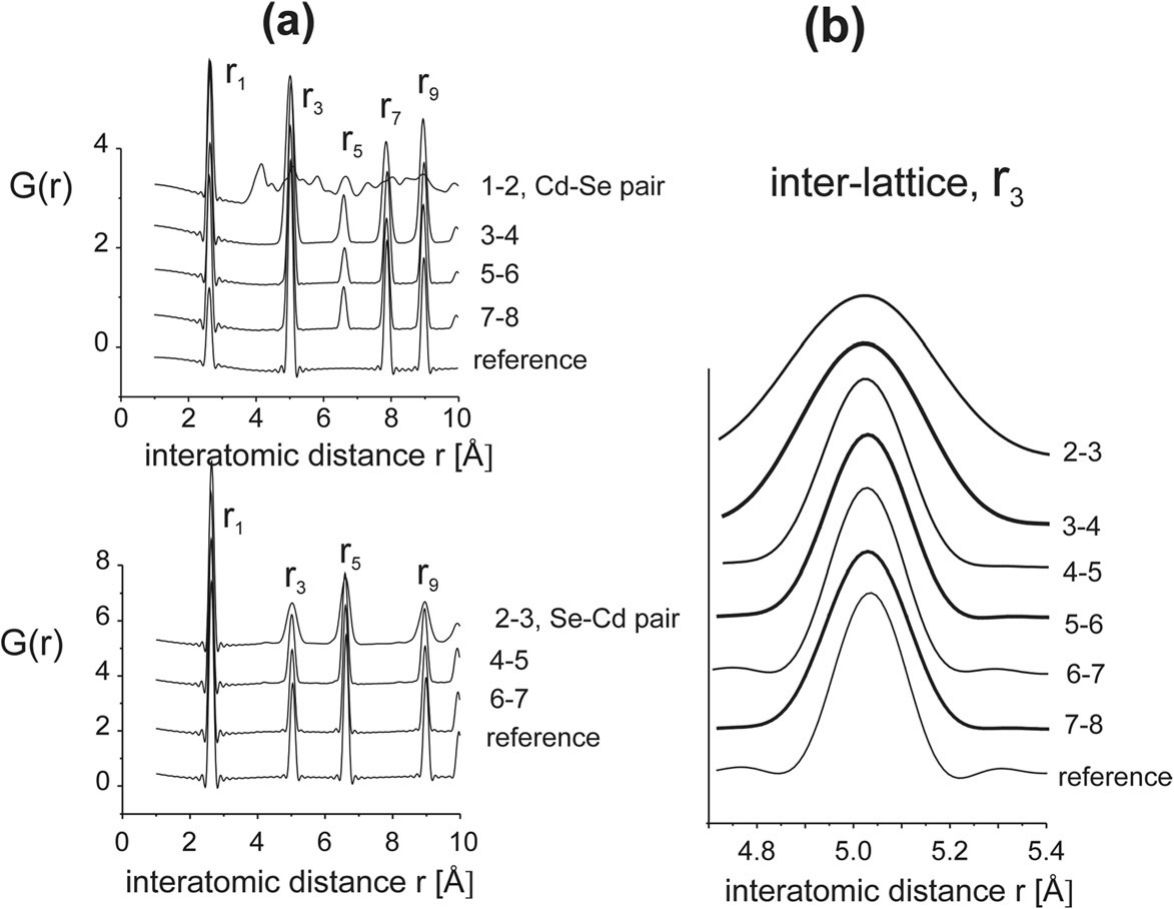
**a** Interatomic distance functions *G*(*r*) for pairs of Cd-Se layers underneath the (111)B end surface. **b** *G*(*r*) lines of Cd-Se *r*_3_ spacing calculated for subsequent pairs of layers for different distances from the surface

The positional disorder observed within individual sub-lattices is also reflected in the dispersion of intra-lattice distances measured between subsequent layers. Figure 7 presents partial *G*(*r*) functions with intra-lattice distances *r*_1_, *r*_3_, *r*_5_, and *r*_7_ calculated for subsequent Cd-Se layer pairs. The first Cd-Se double layer (1–2) is formed from disordered (amorphous-like) Cd layer and the next strongly disordered Se layer. In the corresponding *G*(*r*) curve (Fig. 7a), only one very well-defined sharp peak corresponding to *r*_1_ distance between Cd and Se, and a variety of *r* values with very different lengths, is observed. The *r*_1_ distances measured for the first and also for any pair of Cd and Se atoms in the sample have nearly the same magnitude, which is very similar to that of the perfect CdSe lattice. The influence of surface disordering in the end layer on the internal CdSe lattice is reflected in distances being longer than *r*_1_. Figure 7b shows *r*_3_ peaks in *G*(*r*) calculated for subsequent pairs of layers Cd-Se and Se-Cd. It shows a presence of a positional disordering up to the depth of about 1.5 nm; the peaks are broad near the surface and narrow down starting with the layer pair 4–5.

### Interaction of parallel surfaces

Previously examined 6-nm MD models were thick enough for the surface-related lattice strain to fully relax in the bulk. The relaxation length RL is different for different surfaces and also different for intra- (*r*_2_) and interatomic (*r*_1_ and *r*_3_) distances (Stelmakh et al. 2017). In some cases, it is also different for in-plane and inter-planar distances. The models presently reported are thinner than the relaxation length and the effects originating on the opposite terminating surfaces interfere with each other.

**Fig. 8:**
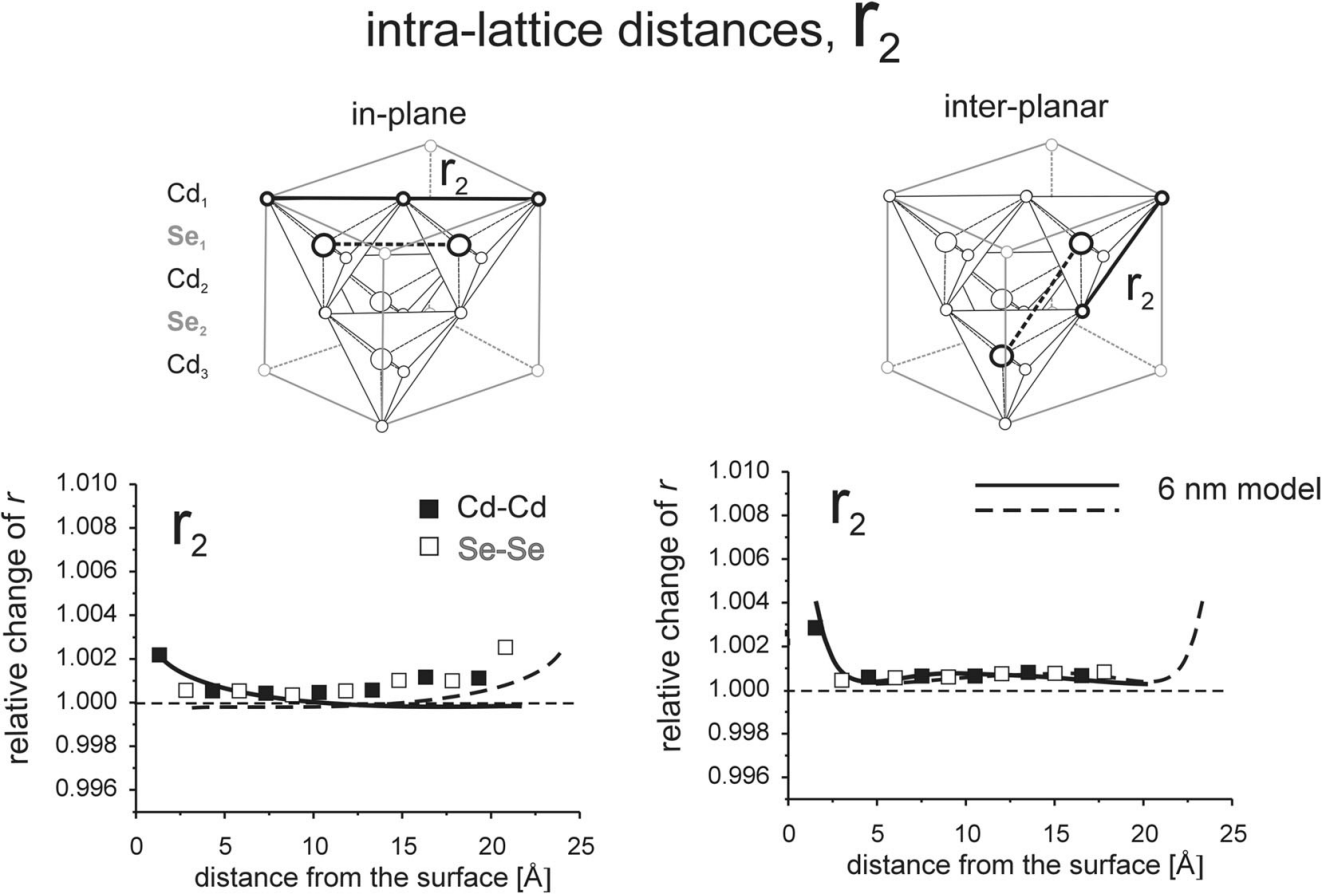
Interatomic distances *r*_2_ calculated for MD model terminated by the (100) surfaces

**Fig. 9:**
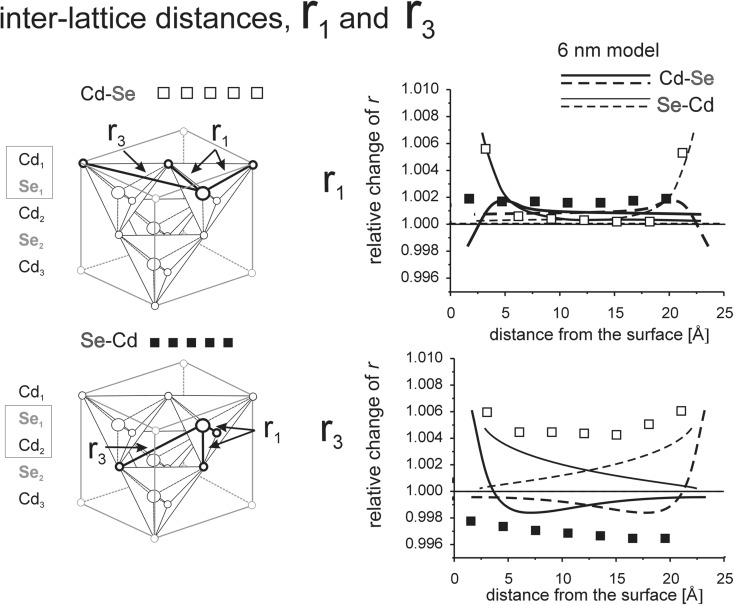
Interatomic distances *r*_1_ and *r*_3_ calculated for MD model terminated by the (100) surfaces. Note: since the end layer is disordered, no distances between the first and second atomic layers are showed in the figure

**Fig. 10:**
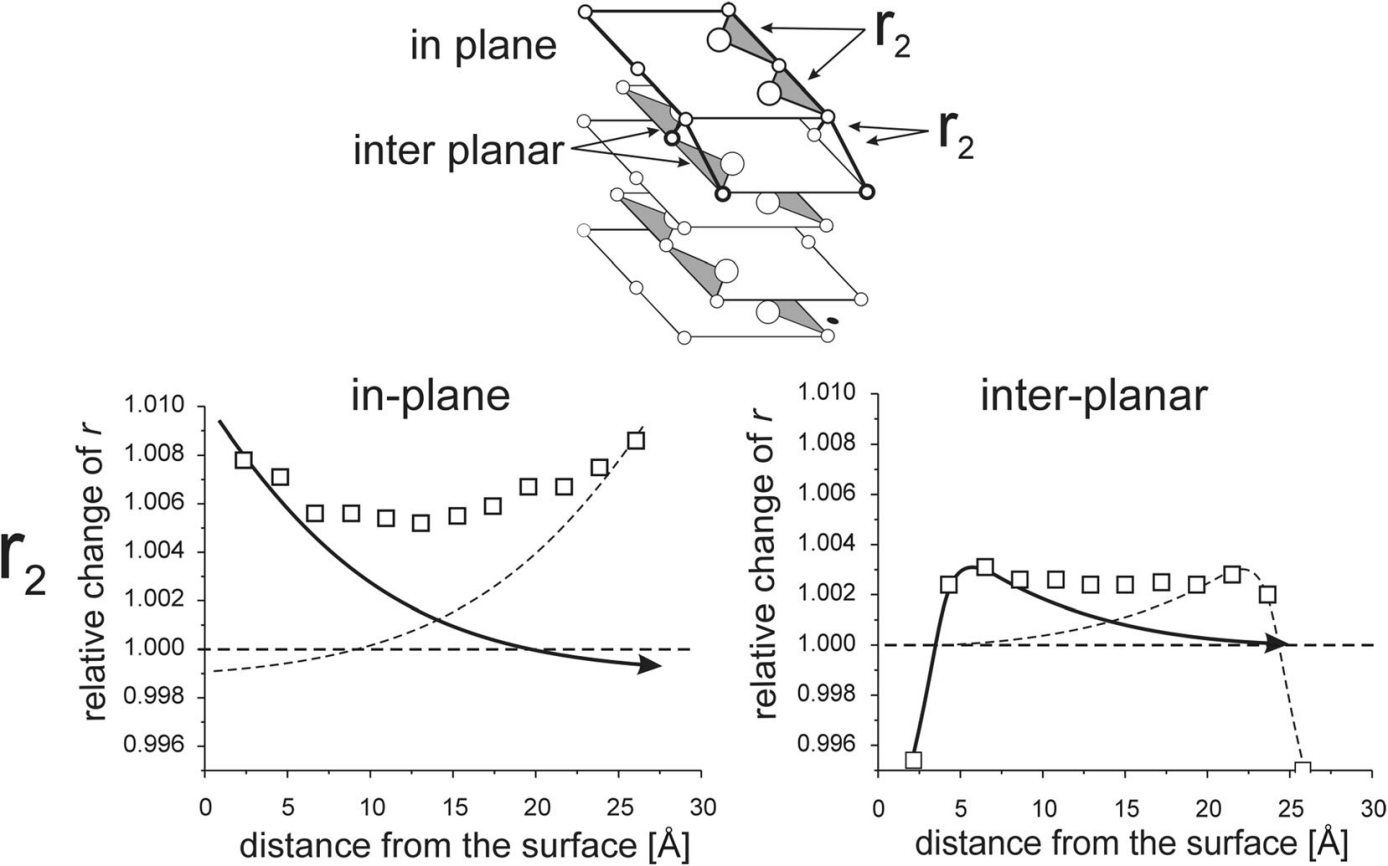
In-plane and inter-planar distances *r*_2_ calculated for MD models terminated by (110) surfaces

**Fig. 11:**
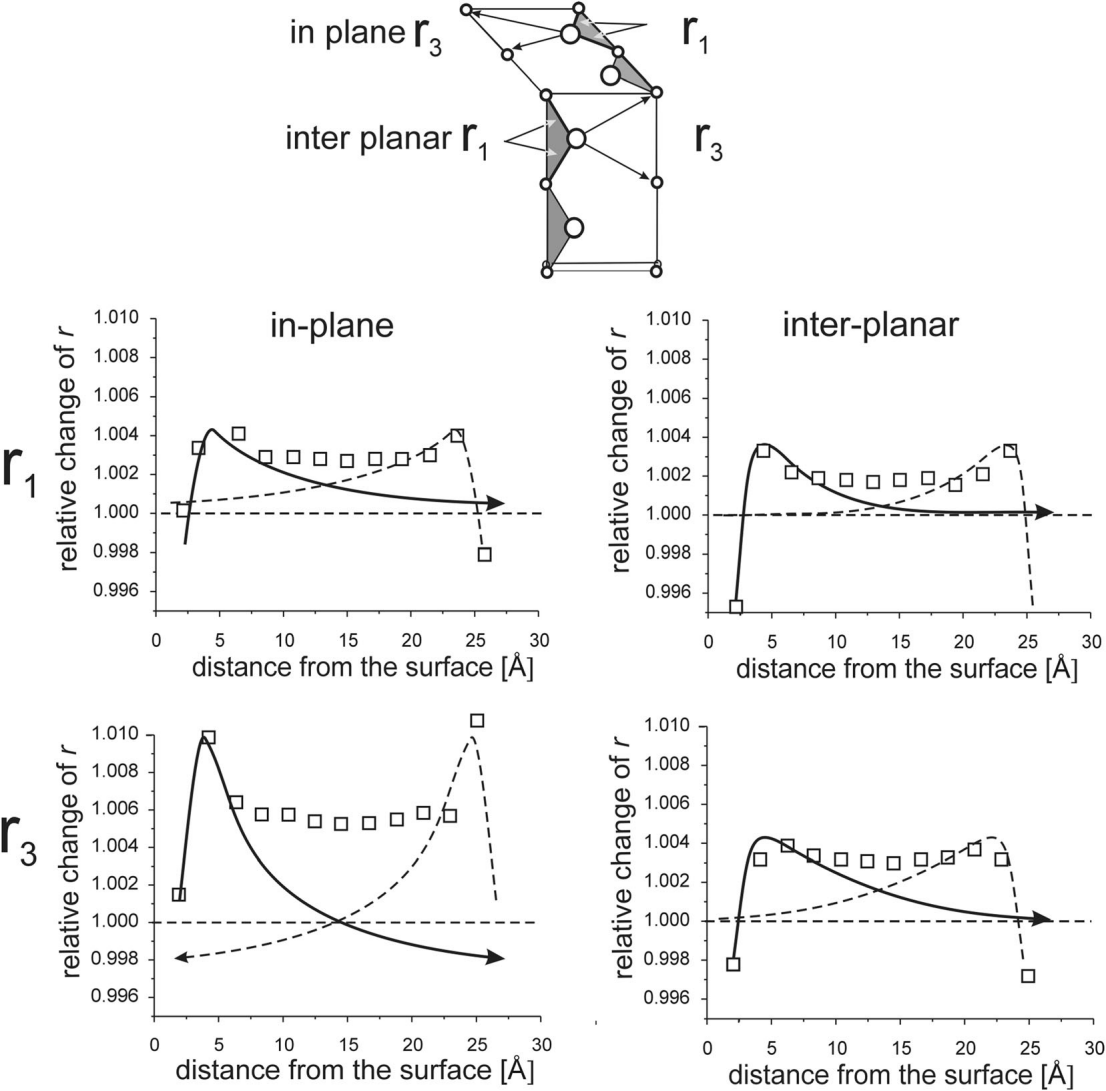
In-plane and inter-planar distances *r*_1_ and *r*_3_ calculated for MD models terminated by (110) surfaces

**Fig. 12:**
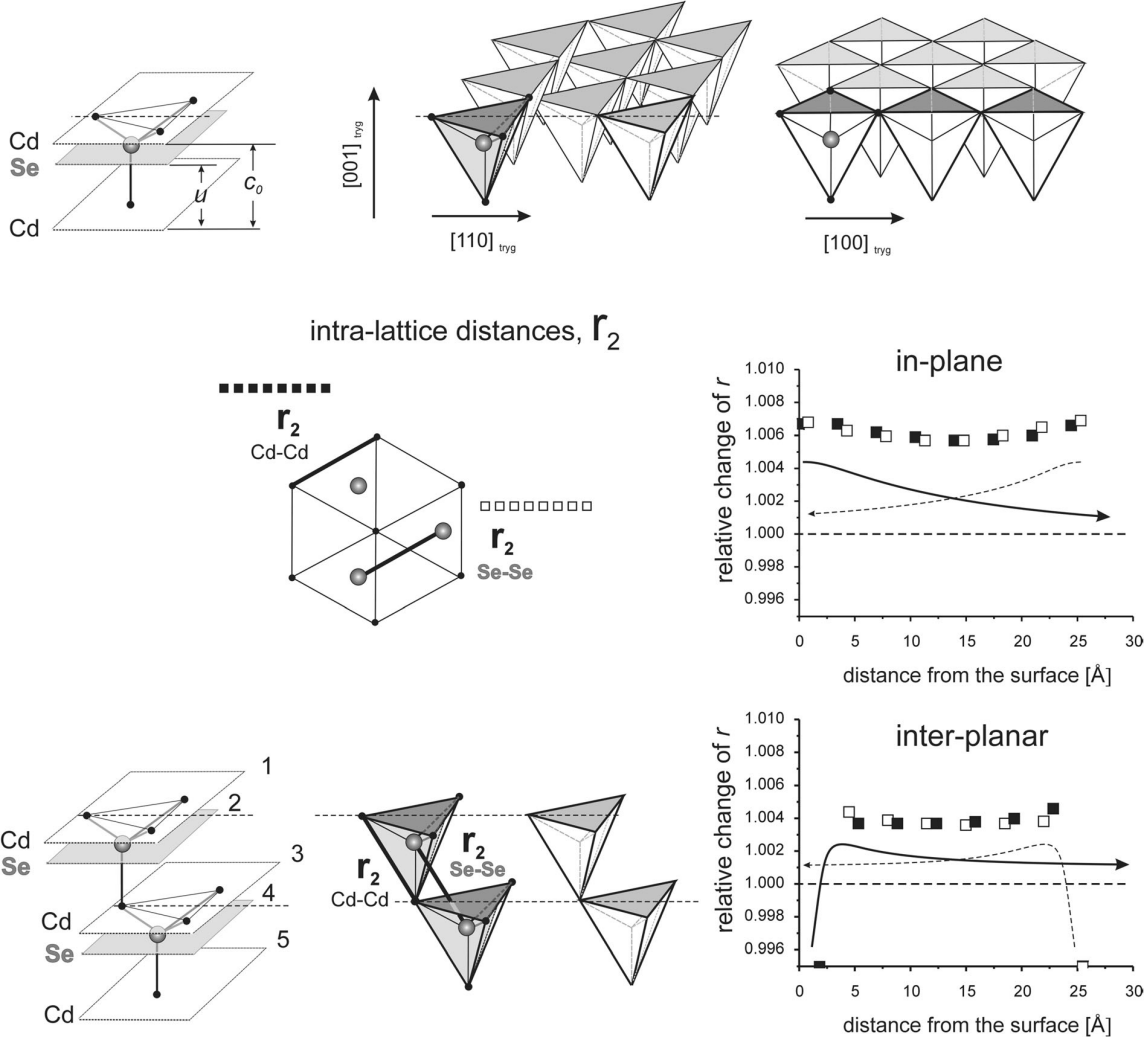
In-plane and inter-planar intra-lattice distances calculated for MD model terminated by (111)A surfaces

**Fig. 13:**
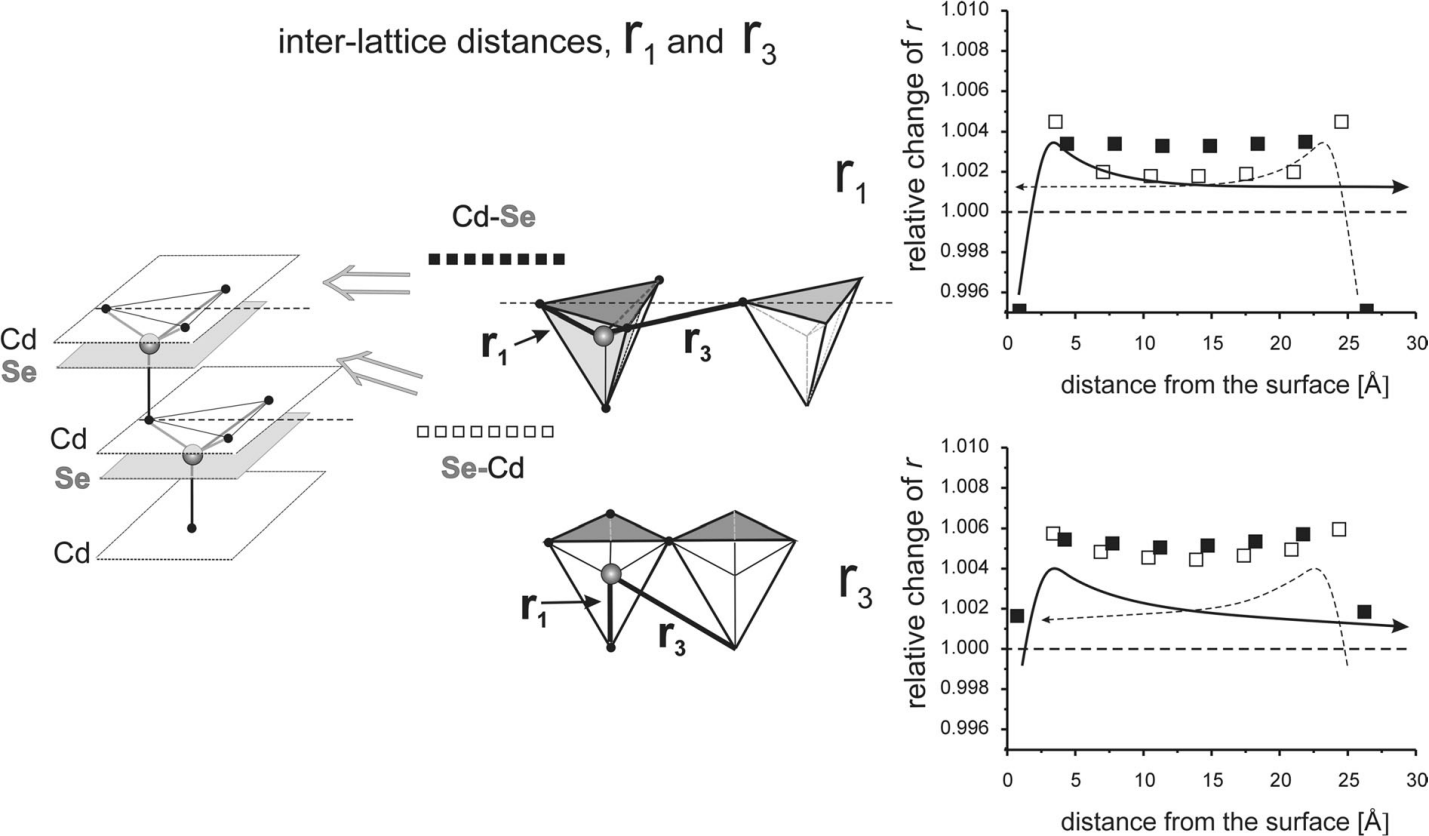
Inter-lattice distances calculated for the MD model terminated by (111)A surfaces

**Fig. 14:**
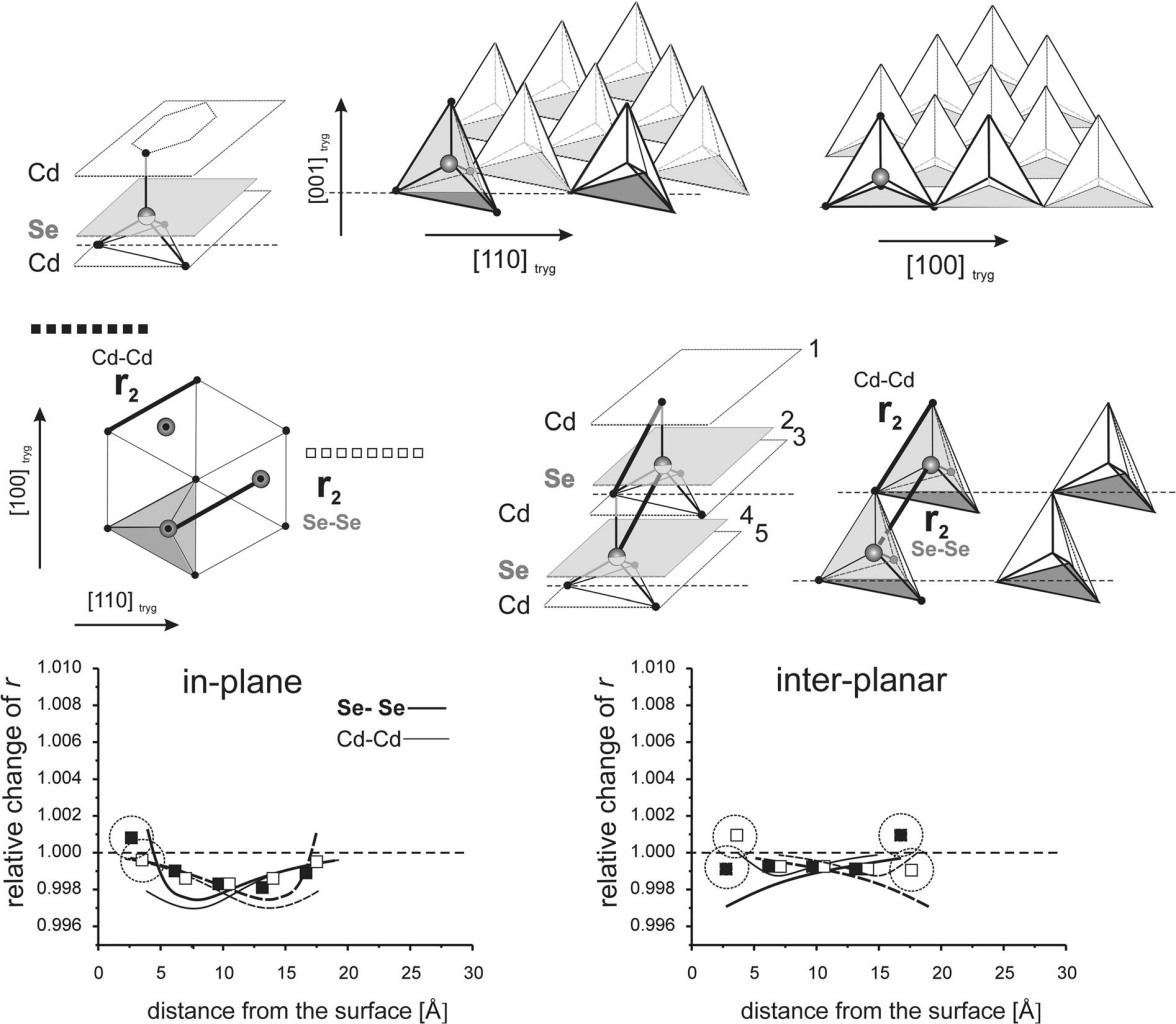
In-plane and inter-planar intra-lattice distances *r*_2_ calculated for the MD models terminated by the (111)B planes

**Fig. 15:**
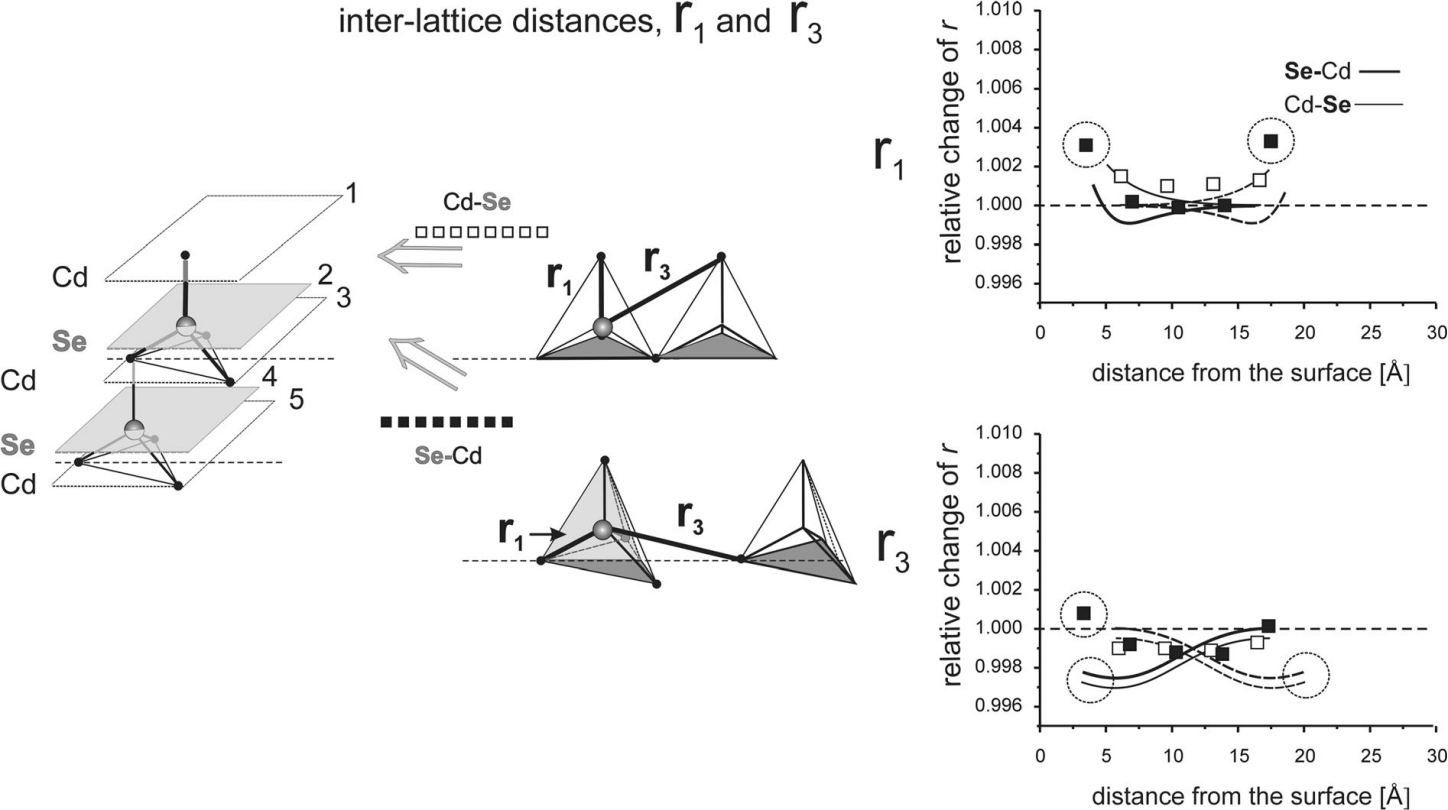
Inter-lattice distances *r*_1_ and *r*_3_ calculated for the MD model terminated by (111)B surface

Interaction between opposite surfaces for thin models is illustrated in Figs. 8, 9, 10, 11, 12, 13, 14, and 15. (Throughout this paper the “*r*_*i*_” values are given as relative to the corresponding value in the perfect lattice.) In addition to the points corresponding to the values of interatomic distances calculated for those models, the results of previous calculations of the surface strain relaxation (6-nm thick models) are shown as solid lines. The broken lines present (hypothetical) changes of the distances that would originate at the opposite (parallel) end layers if the surface effects would not interfere with each other.

#### Interaction of (100) surfaces

The model comprises of nine Cd-Se double layers and is 2.4 nm thick. Relaxation lengths at the (100) surface are as follows: in-plane RL(*r*_2_) ≅ 1 nm, inter-planar RL(*r*_2_) ≅ 0.5 nm, RL(*r*_1_) ≅ 1 nm, and RL(*r*_3_) > 2.5 nm (Stelmakh et al. 2017).

As seen in Fig. 8, *r*_2_ distances, both in-plane and inter-planar, follow the behavior previously observed in thick models. Changes in *r*_2_ length are observed only in the 1–2 surface layers. There is no interaction of the effects originating on the opposite (100) surfaces neither in the Cd nor in the Se sub-lattice. In agreement with the small relaxation length RL(*r*_2_), individual sub-lattices remain undistorted in the bulk.

##### Effect of interaction between (100) surfaces on *r*_1_

Large differences of *r*_1_ calculated for Cd-Se and Se-Cd layer pairs are observed in the first three surface layers, and modest differences are seen in the rest of the model volume (Fig. 9). For thick models, the differences at the surface is also large, but in the inner volume of the model, they are negligible. Different behavior of a thin model is clearly the result of interaction of the effects originating at the opposite surfaces. This interaction leads to a relative shift of Cd and Se sub-lattices in the [001] direction in the bulk part of the model; Se sub lattice gets closer to the Cd-terminated surface.

##### Effect of interaction between (100) surfaces on *r*_3_

A pronounced difference between *r*_3_ distances in Cd-Se and Se-Cd layer pairs is observed in the whole sample volume; *r*_3_ in the Cd-Se pairs increases by approximately 0.4%, while in the Se-Cd pairs, it decreases by approximately 0.2% (Fig. 9). This difference is larger at the surface, which is obviously the result of deformation of the (100) surface in the [110] and [1–10] directions (by − 0.15 and + 0.55%, respectively), c.f. Fig. 2. Also, simultaneous relative shift of Cd and Se sub-lattices (by about 1%) in the [001] direction is observed. While for the thick model, the difference between Cd-Se and Se-Cd *r*_3_ distances diminishes with an increasing distance from the end surfaces; it remains considerable and nearly constant within the whole volume of the nine-layer model. This is a clear indication of the interaction of the effects originating at the opposite sides of the model.

A difference between the interatomic distances *r*_1_ and *r*_3_ calculated for Cd-Se and Se-Cd layer pairs occurs because the fourfold symmetry of (001) planes is lost and also because Cd and Se lattices get shifted with respect to each other in the direction perpendicular to the planes. The length change is comparatively small for *r*_1_ distances, but it is strong for *r*_3_ distances.

The above results explain how the interaction between (100) surfaces leads to deformation of the whole sample volume, which is originally cubic, into orthorhombic type crystal lattice.

#### Interaction of (110) surfaces

The model is 2.6 nm thick and comprises of 13 mixed Cd-Se layers. Relaxation lengths at the (110) surface are as folows: in-plane RL(*r*_1_, *r*_2_, *r*_3_) ≅ 2.5 nm and inter-planar RL(*r*_1_, *r*_2_, *r*_3_) ≅ 2.5 nm (Stelmakh et al. 2017).

Elongation of the in-plane *r*_2_ distances is about 0.8% at the end surfaces and about 0.5% in the middle of the structure (Fig. 10). The value of the 0.8% at the surface is in agreement with the linear expansion of the layer in the [100] and [110] directions, 0.5 and 1%, respectively (c.f. section “Symmetry of (110) surface”). In the thick model, the parent cubic lattice is recovered at the depth of about 2.5 mm. In the thin model, the effects from the opposite surfaces interfere and the elongation of *r*_2_ in the bulk is only slightly smaller than at the surface.

The inter-planar *r*_2_ distances are compressed by about 0.5% just underneath the surface, but they are elongated in the rest of the volume (Fig. 10). In the thick model, we observed a density wave that started with compression at the surface, then expanded the lattice up to the third layer, and then phased out at the depth of about 2.5 nm (Stelmakh et al. 2017). In the thin sample, the interference of two waves originating at the opposite surfaces forms a density wave passing through the whole volume where, in the middle of the structure, the interlayer distance *r*_2_ is still larger by about 0.2% than that in the reference cubic CdSe lattice.

One needs to notice that changes (elongation) of in-plane distances at a given distance from the surface are always about two times larger than elongation of inter-planar distances at the same depth. This obviously follows from the fact that distances between the atoms within layers are longer than between those in adjacent layers, and therefore, the interactions within layers are weaker. Since the strain exerted onto the lattice through surface relaxation tends to be evenly distributed, the local stresses have to be balanced through lattice expansion, which is more pronounced in the directions of weaker bonds.

The changes in inter-lattice distances *r*_1_ and *r*_3_ (Fig. 11) are similar to changes of intra-lattice distances *r*_2_ (Fig. 10). This means that if the model is terminated at both sides by identical 110 surfaces, both sub-lattices, Cd and Se, deform in a correlated manner.

Similarly to changes in intra-lattice distances *r*_2_ presented in Fig. 10, and for the same reasons, the in-plane changes in intra-lattice distances *r*_1_ and *r*_3_, are about two times larger than those calculated for inter-planar distances for the same Cd-Se layer (Fig. 11). During deformation of the (110) surface, both Cd and Se sub-lattices have to deform exactly in the same way. A relative shift of Cd and Se sub-lattices is demonstrated by changes in both *r*_1_ and *r*_3_ distances, where the stronger bonds (*r*_1_) elongate about two times less than the much weaker *r*_3_ bonds. Due to a strong interaction of (110) surfaces, one may expect that in the CdSe crystals with the size of about 5–6 nm will be deformed in the whole grain volume.

#### Interaction of (111)A surfaces

The model comprises eight double Cd-Se layers and is 2.5 nm thick. Relaxation length at the (111)A surface are as follows: in-plane RL(*r*_2_) > 2.5 nm and inter-planar RL(*r*_1_, *r*_2_, *r*_*3*_) > 2.5 nm (Stelmakh et al. 2017).

Figure 12 shows that the effects which originate at both surfaces are nearly perfectly additive; elongation of *r*_2_ distances both in-plane and between the neighboring atomic layers are the sums of the elongations caused by two parallel surfaces (note that inter-planar distance *r*_2_ between the first two layers, 1 and 2, is an exception). The in-plane expansion is nearly two times larger than that between the layers. This is related to weaker bonds between the same atoms within the planes and relatively stronger bonds between neighboring layers belonging to two different sub-lattices, alternatively Cd and Se.

##### Deviation from the perfect *hcp* structure, the *c*_0_/*a* ratio

The difference between *r*_2_ distances (Cd-Cd or Se-Se) measured within individual layers and between two adjacent layers of a given sub-lattice (odd and even: 1 and 3, 2 and 4, etc. in Fig. 12) provides information on the *c*_0_/*a* ratio. For a perfect hexagonal close-packed (*hcp*) structure in-plane and inter-planar *r*_2_ values are equal and the corresponding *c*_0_/*a* equals 0.8167. For our 2.5-nm model, the in-plane *r*_2_ is about 1.007 times larger, and the inter-planar *r*_2_ is about 1.004 times larger than the reference *r*_2_ of the perfect CdSe cubic lattice. The corresponding *c*_0_/*a* ratio is 0.814. That means that the lattice is compressed in the direction normal to the surface relative to the perfect CdSe lattice.

The changes in inter-lattice distances *r*_1_ and *r*_3_ obtained for the 2.5-nm model (Fig. 13) are not a simple sum of changes that originate at both end surfaces. In the thick model, where surfaces are 6.5 nm apart, the values *r*_1_ and *r*_3_ obtained for Cd-Se and Se-Cd pairs of layers are the same (in Fig. 13, they are both represented by the same line) (Stelmakh et al. 2017). In the 2.5-nm model, a distinct difference between *r*_1_ and *r*_3_ distances measured for Cd-Se (1–2, 3–4, etc.) and Se-Cd (2–3, 4–5, etc., c.f. Fig. 12) pairs of layers is found. This behavior results from a relative shift of Cd and Se sub-lattices in the direction normal to the surface.

##### Relative shift of Cd and Se sub-lattices, the *u* parameter

Relative positions of two sub-lattices forming the *hcp* structure are defined by the *u* parameter which describes the relative position of the central atom (e.g., Se) in a tetrahedron (e.g., SeCd_4_) (Fig. 12). In the perfect *hcp* structure, *r*_1_ and *r*_3_ distances are strictly coupled with each other and *u* measured for neighboring layer pairs, i.e., between Cd and Se layers (1 and 2, 3 and 4, etc.) and Se and Cd layers (2 and 3, 4 and 5, etc.) is the same, *u* = 0.75*c*_0_.

The changes in *r*_1_ and *r*_3_ values with a change in distance from the end surfaces for thick models are only slightly different, which means that in this case the *u* parameter is about 0.75*c*_0_; the hexagonal layers which form the structure are strictly close packed. In our thin model, where (111)A surfaces interfere with each other, the relative changes in *r*_1_ and *r*_3_ values, measured for Cd-Se and Se-Cd pairs of layers, do not follow the same line (the open and full squares in Fig. 13 do not overlap). Their divergence demonstrates that the *u* parameter deviates from the 0.75*c*_0_ value. In the bulk part of the model, the difference between *r*_1_ values for the Cd-Se and Se-Cd layer pairs is about 0.2% (1.004–1.002), which corresponds to *u* = 0.7485.

It can be shown that for perfectly periodic *hcp* structure with the *c*_0_/*a* ratio smaller than 0.8167 (as in our model, *c*_0_/*a* = 0.814–0.815, Fig. 12) and *u* ≠ 0.*75c*_0_, the divergence of *r*_1_ values is about 3–4 times smaller than that of the corresponding *r*_3_ values, exactly as observed in Fig. 13.

If the relative shift of Cd and Se sub-lattices was strictly along the threefold axis, the relative changes of the *r*_1_ and *r*_3_ values would be exactly the same. Fig. 13 shows that elongation of the *r*_3_ distances is about two times larger than that of *r*_1_, which means that, together with the relative shift of Cd and Se sub-lattices in the direction normal to the surface, a small in-plane shift in these sub-lattices takes place. Cd and Se sub-lattices get shifted relative to each other in both, perpendicular to the surface and parallel to the surface, directions.

Since relaxation length of the surface strains originating at the (111)A surface, for both intra- and inter-lattice distances, is about 3 nm, the effect of interaction of the (111)A surfaces should be visible in the grains with sizes of about 6 nm and smaller.

#### Interaction of (111)B surfaces

The model comprises six double Cd-Se layers and is 2.1 nm thick. Relaxation length at the (111)A surface are as follows: in-plane RL(*r*_2_) ≅ 2.5 nm and inter-planar RL(*r*_1_, *r*_2_, *r*_*3*_) ≅ 2.5 nm (Stelmakh et al. 2017).

Due to a strong positional disorder at the first two atomic layers, c.f. section “Symmetry of (111)A surface”, both intra- and interatomic distances measured within and between these layers are very much dispersed and the distances calculated for the first three layers are not reliable, c.f. section “Symmetry of (111)B surface” (in Fig. 14, these measurements are shown in circles). The distances *r*_2_ measured in the interior of the six Cd-Se-layer model are quite similar to those measured for the thick, 18-layer model. That indicates a weak interference between (111)B surfaces terminating the model.

##### Deviation from the perfect hcp structure, the *c*_0_/*a* ratio

From the in-plane and interplanar *r*_2_ values (the averages are 0.998 and 0.999, respectively), one can calculate the corresponding *c*_0_/*a* ratio of 0.8175 (= 0.8167 × 0.999/0.998). It is only slightly different from that for the perfect *hcp* lattice. The distortion is in contrast with relatively stronger effect found for the (111)A model, where the *c*_0_/*a* ratio is smaller (0.814–815) than that in the perfect *hcp* lattice, c.f. section “Interaction of (111)A surfaces”.

Figure 15 shows that there is an interaction between the terminal (111)B surfaces, but it is weak and leads to only a small difference between *r*_1_ and *r*_3_ values for both thick and thin MD models. Similarly to the (111)A model, a difference between changes in *r*_1_ and *r*_3_ distances is observed in the whole volume of the model. This difference is only about 0.1% and it corresponds to the *u* parameter which is only slightly larger (about 0.75 × 1.001 = 0.751) than that in the perfect *hcp* lattice. Relaxation of strains at the (111)B surface is related to a strong disorder disturbing the long-range order at the first two atomic layers, while in the bulk part of the model only a small deformation of the *hcp* lattice is observed.

## Summary and conclusions

Properties of a nanocrystal are determined by the atomic structure of both its interior and the surface. At the first approximation some properties of the material are referred to the crystal surface (e.g., adsorption), some to their volumetric part (e.g., optical), and others are a combination of both types of contribution (e.g., compressibility, thermal expansion). It is common to attempt to describe specific physical properties of nanocrystals as being scaled with their dimensions. This approach is correct only if one assumes that the structures of both the surface and of the bulk part of the nanocrystal are invariant with nanocrystal dimensions.

There were numerous approaches to learn about the atomic structure of nanocrystal surfaces using experimental techniques that directly probe interatomic distances like XANES (Hamad et al. 1999), EXAFS (Wu et al. 2007), and NMR (Berrettini et al. 2004). Other such efforts were undertaken through measurements of the elastic properties (Huxter et al. 2009) or studies on the effect of various ligands used for colloidal growth of CdSe nanocrystals on their shape (Nair et al. 2007). Some possible atomic arrangements existing at the surface were suggested to explain experimental observations.

Modeling, both ab initio and MD, has been frequently utilized to investigate CdSe clusters and small crystallites. Most attention have been paid to electronic and phonon structures and far less to the atomic architecture. Atomic positions analysis was limited to the atoms at the very surface and the information on the crystallographic orientation of the analyzed surface was ignored in most cases. Pokrant and Whaley (Pokrant and Whaley 1999) found that the surface Se atoms relax outwards while Cd atoms remain near their original positions. Puzder et al. (Puzder et al. 2004) as well as Botti and Marquez (Botti and Marques 2007) found that surface Cd atoms relax towards the bulk by approximately 0.7 Å, while Se atoms remain in place. Zhu et al. (Zhu et al. 2009) modeled (001) and (111) terminated flat CdSe slabs and found outwards relaxation of Se and inwards relaxation of Cd at (100) surface, while at the (111) surface, some Cd atoms moved in and others moved out. They also reported several specific dimer and tetramer atomic arrangements that appear to be stable on the analyzed surfaces. Only Cherian and Mahadevan (Cherian and Mahadevan 2008) reported gradual increase of the bond length between the center and the surface in small CdSe clusters. In general, available information on the lattice deformation in CdSe nanocrystals is scarce, inconclusive, and often contradictory. Change of the bond lengths and lattice rearrangement is always found at the top layer, but its influence on the underlying crystal lattice is hardly discussed.

Knowledge on structural processes that may occur at the grain surface is desired to verify the proposed models and correlate them with specific physical properties of nanocrystalline materials. This work shows a need for verification of “general considerations” of thermodynamic properties like the melting point temperature (Cherian and Mahadevan 2008) or the “internal pressure” (Fu et al. 2017) which are routinely considered as size-dependent properties of nanocrystal. Without taking into account a specific surface structure of individual nanocrystals, many properties of real nanomaterials cannot be satisfactorily explained.

As a result of crystal truncation (surface formation), some of the bonds of the surface atoms get broken and the remaining ones change the length of their bonds in order to accommodate the new environment that leads to a formation of strain. Relaxation of the surface strains is necessary to establish a new equilibrium state corresponding to the minimum free energy of the whole ensemble of atoms constituting the crystal. It is realized through (i) changes of the nearest neighbor coordination (i.e., formation of new bonds), (ii) an appearance of disordering, and (iii) changes in the lengths of the existing bonds.

In a thick CdSe platelet, a surface terminating the volume changes the parent crystal lattice underneath down to the depth of 2–3 nm (Stelmakh et al. 2017). The bond length changes at the surface planes and between atomic planes underneath it are typically between 0.2 and 0.5%, but in some cases, they reach even 1%. The strongest strains occur at the surface and they gradually diminish with an increase in the distance from the surface until the parent (perfect) lattice is fully recovered.

In the models examined in the present work, only one dimension was in the nano-range, which allowed to determine what kind of deformation different truncating surfaces introduce into the crystal lattice. In real 3-D nanocrystals, the strains originating at different surfaces interfere with each other. In this work, we examined and quantified the effect of interference of strains originating at two opposite parallel surfaces of the same type terminating a platelet-like crystal. We showed that the observed structural changes concern not only the lengths of interatomic bonds, but they may also lead to a loss of the long-range order at the end atomic layer. It may also result in a reduction of the symmetry of the crystal lattice. The opposite surfaces interfere with each other when the sum of their surface strain relaxation lengths is larger than the distance between them. In a nanocrystal, the whole volume is affected by surface strains, regardless of which of the four types of surface examined in this work confines the crystal volume.

In the models examined in the present work only one dimension was in the nano-range, which allowed to determine what kind of deformation different truncating surfaces introduce into the crystal lattice. In real 3-D nanocrystals, the strains originating at different surfaces interfere with each other. In this work, we examined and quantified the effect of interference of strains originating at two opposite parallel surfaces of the same type terminating a platelet-like crystal. We showed that the observed structural changes concern not only the lengths of interatomic bonds, but they may also lead to a loss of the long-range order at the end atomic layer. It may also result in reduction of the symmetry of the crystal lattice. The opposite surfaces interfere with each other when the sum of their surface strain relaxation lengths is larger than the distance between them. In a nanocrystal, the whole volume is affected by surface strains, regardless of which of the four types of surface examined in this work confines the crystal volume.

The strain relaxation processes occur differently in structures with different surfaces and they have different influences on the inner part of the crystal lattice:

- Symmetry:
  1. At the (100) surface layer, the long-range order is lost. The fourfold arrangement of atoms in the subsequent layers turns into orthorhombic-type arrangement and, thus, the whole lattice becomes rhombohedral.
  2. At the (110) layer, the symmetry of the original atomic arrangement is preserved, but in-plane dimensions are changed. As a result, the lattice underneath the surface is also deformed, and the cubic symmetry of the whole volumetric part of the grain is reduced to orthorhombic-like lattice.
  3. At the (111)A surface, the original threefold symmetry of the end atomic layer is preserved. The threefold symmetry is preserved also in the whole crystal volume, although the *c*/*a* ratio and the relative shift of Cd and Se sub-lattices, *u*, are changed relative to the original *hcp* lattice.
  4. At the (111)B terminal, atomic layer the long-range order is lost and so is the trigonal symmetry. The next two atomic layers underneath the disordered surface are strongly deformed. In the subsequent layers, the threefold symmetry is recovered, although both the *c*/*a* ratio and the *u* parameters are changed relative to the perfect *hcp* reference lattice.
- Disordering:

A positional disordering (deviation of equilibrium atomic positions from perfectly periodic arrangement) is present in all models under examination. It is always the largest at the surface layer and decreases with an increase in the distance from the surface.

An appearance of the strongest disordering is accompanied by a loss of long-range order that occurs at (100) and (111)B surfaces. These surfaces show the atomic order which is intermediate between crystalline and amorphous-like structures. The disordering that is present at the (100) surface has only a small effect on the disorder appearing in next layers, but disordering at the (111)B surface has a strong effect on individual hexagonal layers in the entire grain volume.

- Change of bond lengths—internal strains:

A local change of interatomic intra-lattice distances (e.g., an expansion) always leads to a reciprocal “response” of the crystal lattice (a compression) that compensates the strains associated with the primary changes. Our present results show that an appearance of disordering is an alternate mechanism of strain relaxation.

Note that strains are related to intra-lattice bonds *r*_2,_ which decide on the lattice expansion or compression, while any changes in bonds between sub-lattices have no direct effect on the lattice density.

The largest changes in intra-lattice bond lengths are observed in the models where the smallest disordering is observed. In models with (110) and (111)A surfaces, the long-range order is well preserved and the changes in the lengths of interatomic bonds are the largest, up to 1% at the surface (Figs. 10, 11, 12, 13). The strongest disordering occurs in the model with the (111)B surfaces where local changes in bond lengths are only about 0.2–0.3% (Figs. 14 and 15). Similarly, in models with the (100) surfaces where the long-range order is lost at the terminal atomic layer, only small changes in the lengths of the interatomic bonds within Cd and Se sub-lattices are observed (Figs. 8 and 9).

MD calculations reported here were performed with the assumption that the CdSe nanocrystals are not passivated, i.e., they placed in a vacuum. Other types of environment, gases, liquids, organic ligands, or a solid coating may have a strong effect on the surface and consequently on the bulk structure. That might have a pronounced effect on the internal structure of materials and, consequently, affect their physical properties. Calculations of the effects of the CdSe environment (adsorbates, shells) on the nanocrystal structure are currently under investigation.
